# MBL-TransUNet: Enhancing Mesostructure Segmentation of Textile Composite Images via Multi-Scale Feature Fusion and Boundary Guided Learning

**DOI:** 10.3390/ma18061215

**Published:** 2025-03-09

**Authors:** Hang Qi, Aiqing Ni, Yuwei Feng, Yunsong Peng, Bin Yang, Guo Li, Jihui Wang

**Affiliations:** 1 School of Material Science and Engineering, Wuhan University of Technology, 122 Luoshi Road, Hongshan District, Wuhan 430070, China; 331055@whut.edu.cn (H.Q.); 222524@whut.edu.cn (Y.F.); pysjiaoda@163.com (Y.P.); jhwang@whut.edu.cn (J.W.); 2 State Key Laboratory of Advanced Technology for Materials Synthesis and Processing, Wuhan University of Technology, 122 Luoshi Road, Hongshan District, Wuhan 430070, China; 3 Luoyang Ship Material Research Institute, 169 Binhe South Road, Luolong District, Luoyang 471039, China; liguo_whut@163.com

**Keywords:** fabrics/textiles, microcomputed tomography, deep learning, image segmentation

## Abstract

Accurate segmentation is essential for creating digital twins based on volumetric images for high fidelity composite material analysis. Conventional techniques typically require labor-intensive and time-consuming manual effort, restricting their practical use. This paper presents a deep learning model, MBL-TransUNet, to address challenges in accurate tow-tow boundary identification via a Boundary-guided Learning module. Fabrics exhibit periodic characteristics; therefore, a Multi-scale Feature Fusion module was integrated to capture both local details and global patterns, thereby enhancing feature fusion and facilitating the effective integration of information across multiple scales. Furthermore, BatchFormerV2 was used to improve generalization through cross-batch learning. Experimental results show that MBL-TransUNet outperforms TransUNet. MIoU improved by 2.38%. In the zero-shot experiment, MIoU increased by 4.23%. The model demonstrates higher accuracy and robustness compared to existing methods. Ablation studies confirm that integrating these modules achieves optimal segmentation performance.

## 1. Introduction

Composites reinforced with continuous fibrous fabrics exhibit a complex multi-scale structure [1], which plays a pivotal role indetermining the mechanical properties of the final composite components. However, the relationship between the macroscale behavior of composites and their internal architecture remains poorly understood yet, mainly due to the the intricate meso- and micro-structures of the reinforced fabrics. A prevalent method to investigate this link involves destructive dissection of the sample, followed by the two-dimensional imaging techniques to analyze its internal structure [2]. This approach necessitates the slicing of the sample at predetermined intervals, yielding only partial information regarding the material’s internal features and resulting in a fragmented comprehension of the overall structure. To address this deficiency, three-dimensional imaging techniques have been employed recently, with Micro-Computed Tomography (Micro-CT) emerging as a notable example [3,4,5]. Micro-CT is a non-destructive imaging technique that captures two-dimensional X-ray projections of a specimen from multiple angles. These projections are subsequently reconstructed into a three-dimensional volumetric representation using advanced algorithms, such as Filtered Back Projection (FBP), Algebraic Reconstruction Techniques (ART), and mordern deep learning-based approaches.

In fiber-reinforced composites, accurate segmentation of Micro-CT images is a critical step in transforming these volumetric data into digital twin models, which are indispensable for numerical simulations. The precision of segmentation directly influences the reliability of the predictions regarding the mechanical behavior and performance of composite materials. Thus, achieving high segmentation accuracy is essential for establishing a robust connection between imaging data and material property analysis.

Over the years, various segmentation algorithms have been applied to analyze 3D image sequences and distinguish fiber tows. These include edge detection segmentation [6], threshold segmentation [7], region growing segmentation [8], watershed segmentation [9], and active contour model segmentation [10]. However, these traditional methods often require substantial manual intervention and are prone to segmentation errors, limiting their practical applicability in complex applications. This underscores the pressing need for more advanced and automated segmentation techniques to address these challenges and enable more precise analysis.

**Figure 1:**
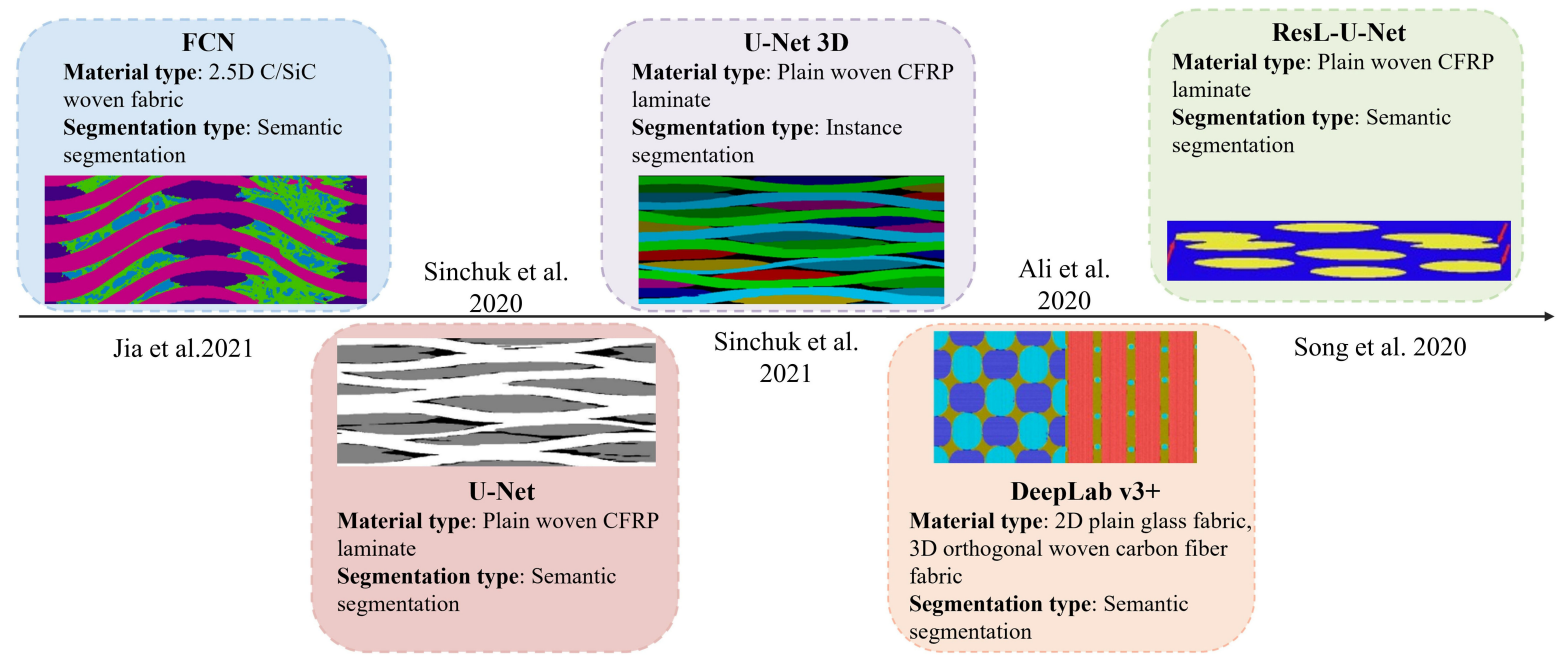
The Evolution of Deep Learning-Based Segmentation Models in Composite Materials Since 2015. Semantic segmentation assigns a categorical label to each pixel, treating all objects within the same class as a single entity, such as fiber tows and the matrix. In contrast, instance segmentation not only categorizes pixels but also distinguishes individual instances within the same class, exemplified by the distinct identification of each fiber bundle.The images shown in the figure are sourced sequentially from references [15,17,18,20,21].

In recent years, deep learning techniques have been widely applied to the segmentation and reconstruction of 3D images [11]. Convolutional neural networks (CNNs) [12], in particular, have gained significant attention due to their ability to process structured grid-like data and autonomously extract hierarchical features. This capability makes them exceptionally well-suited for complex image analysis tasks, markedly improving the efficiency and accuracy of feature identification, including applications such as pathological tissues [13]. Since 2015, these advanced image segmentation methods has been progressively adopted in the field of composites. Figure 1 provides a chronological overview of deep learning models utilized in the analysis of composite materials, organized by their development timeline rather than the specific timing of their application.

Long et al. [14] introduced the Fully Convolutional Network (FCN) in 2015, establishing a foundational framework for image segmentation methods. Subsequently, Jia et al. [15] utilized threshold segmentation algorithms from OpenCV for pixel classification in Micro-CT images with regular microstructures. These regular microstructures refer to the symmetric arrangement of warp and weft yarns in 2.5D woven fabrics, where fiber tows are periodically interlaced with uniform spacing to form a repetitive, organized pattern. The classification outcomes were used as ground-truth labels to train a proposed multi-decoder FCN for segmenting XCT images of complex internal microstructures in 2.5D woven fabrics. Their results demonstrated strong alignment between predictions and experimental findings.

Noise introduces ambiguity at object boundaries and in uniform regions, making it harder to separate features from the background. This significantly reduces segmentation accuracy. To solve the problem of noise interference, Ronneberger et al. [16] improved the FCN architecture in 2015 and proposed the U-Net convolutional network. U-Net reduces the impact of noise using its symmetric encoder-decoder structure. This design allows the model to capture both low-level and high-level features. In addition, the skip connections in U-Net directly transfer fine spatial details from the encoder to the decoder. This helps the model recover from noise and achieve more accurate segmentation results. Sinchuk et al. [17] applied both traditional image segmentation algorithms and the U-Net deep learning algorithm to extract and reconstruct the warp and weft yarns in woven composites. The U-Net approach achieved the highest segmentation accuracy. Building on this, Sinchuk et al. [18] proposed two instance segmentation methodologies for carbon fiber composites: an interpolation-based approach leveraging interlaminar matrix layers and a U-Net 3D method [19] to generate the optimal inputs for watershed transformation. Compared to geometrical methods, deep learning approaches proved more robust with non-ideal inputs, such as fiber bundles with large contact areas or small orientation variations. The maximum segmentation error using deep learning was 0.81%, outperforming the 1.12% error of geometrical methods. Furthermore, the adaptability of deep learning models to diverse training datasets and input parameters enables superior performance optimization compared to traditional methods.

In 2018, Chen et al. [22] introduced the DeepLab V3+ model, incorporating innovations like atrous convolutions, multi-scale atrous spatial pyramid pooling [23], and global context information fusion. These advancements optimized contextual information utilization and segmentation accuracy, achieving superior performance in semantic segmentation tasks. Ali et al. [21] futher modified the DeepLab V3+ network by using ResNet18 as the backbone, achieving a segmentation accuracy of 91% on 2D plain weave glass fabrics, significantly outperforming k-NN’s 71% accuracy.

While these models are built on Convolutional Neural Networks (CNNs), which are central to deep learning in image processing, they exhibit limitations in capturing global semantic interactions and long-range dependencies. CNNs are adept at extracting local spatial features but rely on multiple convolutional layers to expand the receptive field, which often proves insufficient for global context modeling. This limitation is particularly pronounced in fiber segmentation, where accurately distinguishing fiber tows in complex woven structures demands comprehension of global continuity and spatial relationships. Capturing such dependencies is essential for resolving intricate interlaced patterns where fiber tows span multiple regions.

Transformers, known for their self-attention mechanisms, excel at modeling global context and capturing long-range dependencies, effectively bridging the gap left by CNNs in handling both global and local features simultaneously. The Vision Transformer (ViT) [24] was the first to introduce this architecture to the visual domain, leveraging global self-attention for impressive results. However, as image resolution increases, ViT’s computational cost escalates, and it struggles to capture fine-grained local details. Swin Transformer [25] addresses this by using local window attention, reducing complexity and improving local feature capture but sacrificing some global context modeling capabilities.

TransUNet [26] integrates the global attention mechanisms of Transformers with the precise localization capabilities of U-Net, effectively balancing global context modeling and local feature extraction. This hybrid architecture is particularly effective for complex tasks, such as medical image segmentation [27], making it a promising approach for composite fabric image segmentation. Table 1 summarizes the advantages and limitations of the aforementioned algorithms in the context of composite fabric image segmentation.

The objective of this study is to introduce a TransUNet-based framework for segmenting 3D image sequences of fiber-reinforced composites in CT scans, leveraging advancements in automated deep learning architectures. To enhance the model’s representational capacity, a multi-scale feature fusion module, integrated with a boundary enhancement module, was developed to expand the receptive field and capture more intricate features. Additionally, a novel Boundary-guided Learning Module (BLM) was designed to autonomously learn boundary features, further improving segmentation accuracy. Finally, the BatchFormerV2 module was incorporated between CNN and Transformer architectures to enable cross-learning among batch samples, thereby enhancing the model’s generalization capacity.

This paper is organized as follows: Section 2 delivers a thorough exploration of the comprehensive data processing pipeline, including data acquisition, preprocessing, manual segmentation, and data augmentation. Section 3 provides a in-depth description of the TransUNet-based automation framework, detailing the multi-scale feature fusion module, BLM, BatchFormerV2 module, loss function, performance metrics, and the approach for the automated segmentation of connected fiber bundles. Section 4 delineates the methodologies for model training and evaluation, accompanied by a discussion of the results. Conclusively, Section 5 provides a summary of the principal contributions of this research.

## 2. Data Processing

### 2.1. Data Acquisition

**Figure 2:**
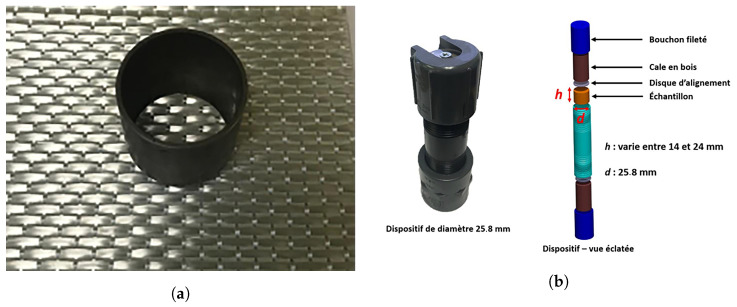
(**a**) The actual sample of the fabric used. (**b**) Schematic representation of the device with an inner diameter of 25.8 mm [28].

In this study, a preform consisting of eight plies of glass non-crimp fabric was scanned using Micro-CT. The actual sample images of the preform and the compression device used in Hilal’s study are shown in Figure 2, with the image data sourced from the thesis by Hilal [28]. Scanning was conducted at a resolution of 0.022 mm (voxel size 0.022 mm). This setup enabled high-precision capture of microstructural features in three-dimensional space. The scanned volume measured 1675 × 1675 × 1196 voxels. The source-to-object and source-to-detector distances were 111.2 mm and 1010.94 mm, respectively, with a detector resolution of 0.2 mm/pixel. This spatial resolution directly influenced the clarity and sharpness of projection images.

A total of 2634 projection images were captured at an angular increment of 0.1367 degrees. Of these, 704 high-quality images from the central region were selected for analysis. Subsequently, the same sample underwent compaction, increasing its fiber volume fraction from 54% to 60%, followed by a second Micro-CT scan. These scans corresponded to two different fiber volume fractions of the same specimen. They were used to construct two distinct datasets.

### 2.2. Pre-Processing and Manual Segmentation

Due to the absence of publicly available annotated datasets, a novel image segmentation training dataset has been developed and will be released as an open resource. The open-source platform Fiji/ImageJ [29] was used during preprocessing. It adjusted the contrast and brightness of image slices to enhance the distinction between fiber bundles and the background. Subsequently, the thresholding function was applied to set background grayscale values to zero, effectively minimizing potential noise. This thresholding operation is expressed as follows:

(1) $$I_{\mathrm{processed}}=\left\{\begin{array}{cc} 0, & \mathrm{if}\;I_{\mathrm{original}}<T\\ I_{\mathrm{original}}, & \mathrm{if}\;I_{\mathrm{original}}\geq T \end{array}\right.$$

$I_{\mathrm{original}}$ represents the initial pixel value, *T* denote the threshold, which was set to 201 in this study. Pixels with intensities below *T* were assigned a value of zero to effectively suppress background noise.

**Figure 3:**
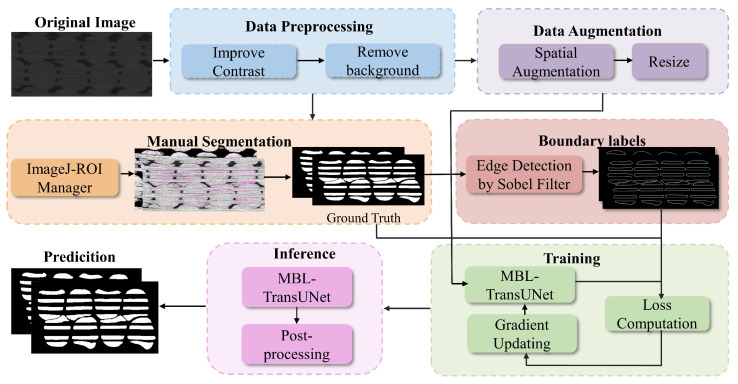
The automated framework designed for segmenting 3D image sequences of textile-reinforced composites.

To generate accurate training labels, the preprocessed images were manually segmented in Fiji/ImageJ, as shown in Figure 3. Special attention was given to annotating and separating contact regions in cross-sections. This step enabled the model to accurately distinguish adjacent fiber bundles. After segmentation, edge detection was applied using the Sobel operator to extract boundary labels from the manually segmented images. These boundary labels explicitly delineated the edges of fiber bundles, facilitating shape-guided training [30]. Both the edge-detected boundary labels and the manually segmented labels were then binarized. Each pixel was assigned to either the foreground (fiber) or background. This ensured compatibility with the loss function during training.

### 2.3. Data Augmentation

To expand the training dataset, binary labels and their corresponding images were subjected to data augmentation techniques. These included random rotation, flipping, color adjustment, and resizing to maintain consistency between image-label pairs and enhance dataset diversity [31].

Data augmentation was applied randomly to the original dataset without altering the total number of samples. Each image had a 50% chance of undergoing either random rotation or flipping, which were applied mutually exclusively. Color enhancement involved brightness adjustment (±20%) and contrast adjustment (±20%). These were applied independently with a 10% probability. If the image dimensions did not match the target size, resizing was applied. Random rotation improved the model’s robustness to variations in fiber orientation. Flipping introduced additional variability through mirroring. Color enhancement simulated diverse lighting conditions, improving the model’s adaptability. Resizing ensured the model could handle images of varying scales. These techniques collectively increased the variety of input data. They effectively mitigated overfitting and enhanced the model’s generalization capability.

Finally, the augmented dataset and segmented labels were divided into training, validation, and test sets. The division was done in an 8:1:1 ratio. Out of a total of 704 images, 564 were allocated to the training set, 70 to the validation set, and 70 to the test set.

## 3. Methodology

Boundary segmentation errors are common in the image segmentation of fiber-reinforced composites. These errors occur particularly when fiber bundles are in close proximity. Deep learning methods outperform traditional algorithms in robustness and accuracy. However, achieving precise boundary segmentation and strong generalization remains challenging. To address these issues, this paper proposes the Multi-scale Boundary-guided Learning TransUNet (MBL-TransUNet) model.

**Figure 4:**
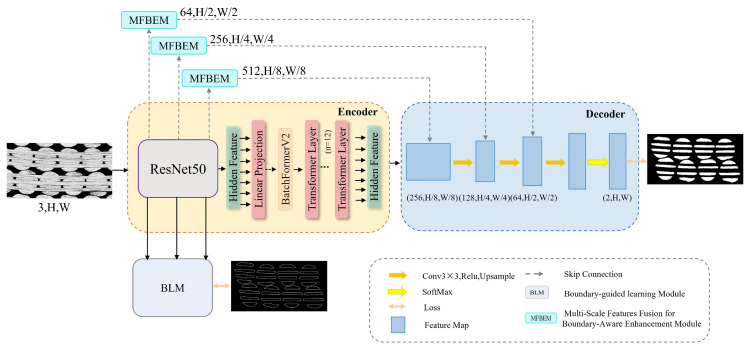
Schematic representation of the MBL-TransUNet.

The proposed model is built on the TransUNet framework. It retains the ResNet50 backbone network to utilize its strong feature extraction capabilities. The model incorporates multi-scale feature fusion modules, a BLM, and the BatchFormerV2 module. The overall network architecture is illustrated in Figure 4. Arrows in the figure represent the direction of forward propagation. They illustrate the flow of feature maps. For clarity, the backpropagation directions are intentionally excluded.

Figure 3 illustrates the proposed framework, which consists of six key stages: image preprocessing, manual segmentation, boundary-guided segmentation, data augmentation, training, and evaluation. The ground-truth labels, obtained through manual segmentation, are used during training for pixel-wise classification, ensuring differentiation between fibers and the background. Boundary labels are derived from the ground-truth labels using the Sobel operator and provide additional supervision. They assist the model in capturing the shapes and edges of fiber bundles. These two types of labels work together, with backpropagation optimizing the model for both tasks. In the evaluation stage, the model generates segmentation predictions, which are then evaluated to assess its performance.

The proposed model was implemented using the PyTorch deep learning framework with Python 3.8. The computational environment consisted of a 12th Gen Intel(R) Core(TM) i5-12400F CPU, an NVIDIA GeForce RTX 4060 Ti GPU, and the Ubuntu 18.04.1 (64-bit) operating system.

### 3.1. Multi-Scale Feature Fusion for Boundary Enhancement Module (MFBEM)

**Figure 5:**
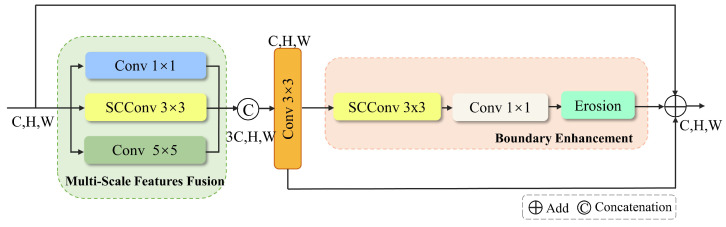
Schematic diagram of the Multi-scale feature fusion and Boundary Enhancement Module. Add and concatenation are common operations in neural networks for combining feature maps. Add sums multiple feature maps element-wise, producing an output feature map with the same dimensions as the input. This operation effectively combines features while preserving information integrity. Concatenation concatenates feature maps along the channel or spatial dimension, increasing the output feature map’s channel count. It is commonly used to merge information from different sources, generating a more informative feature map while changing the output dimensions.

Multi-scale Feature Fusion for Boundary Enhancement Module (MFBEM) is designed to improve feature extraction and enhance boundary detection accuracy. As shown in Figure 5, this module comprises two main components: Multi-Scale Feature Fusion (MF) and Boundary Enhancement (BE). To effectively extract multi-scale features, convolutional kernels of varying sizes are applied to the input features. Specifically, Conv 1 × 1 captures localized details with minimal spatial complexity, making it ideal for refining pixel-level features. SCConv 3 × 3 leverages Spatial and Channel Reconstruction Convolution (SCConv) [32], reducing spatial and channel redundancy while preserving rich feature representations. Conv 5 × 5 processes features at a larger receptive field, providing contextual information [22].

The outputs from these convolutions are concatenated along the channel dimension to fuse features across multiple scales. This fused feature map is then processed through a Conv 3 × 3 layer to integrate and refine features, enhancing feature consistency. The refined feature map is subsequently passed into the boundary enhancement module, which includes SCConv 3 × 3 for enhancing feature representation, Conv 1 × 1 for compressing the channel dimension and highlighting key features, and morphological erosion [33], a post-processing operation for noise suppression, boundary smoothing, and improved boundary detection.

Finally, the input feature map, the processed Conv 3 × 3 feature map, and the output from the boundary enhancement module are fused using an element-wise sum operation. The input feature map preserves global information, the convolutional feature map captures local details, and the boundary enhancement module improves boundary localization. These complementary feature representations are integrated to enhance segmentation performance, particularly in complex image regions with fine structural details and ambiguous boundaries.

### 3.2. Boundary-Guided Learning Module (BLM)

The encoder in TransUNet combines multiple convolutional layers with Transformers for feature extraction. However, it relies on frequent upsampling to restore the original image resolution. This process causes significant spatial information loss [34].

This problem is especially pronounced in textiles with repetitive, periodic structures. These structures have two types of interfaces: the heterogeneous interface between fiber bundles and the matrix, and the homogeneous interface between adjacent fiber bundles. The latter poses a particular challenge due to the high similarity in material properties and grayscale intensity, resulting in ambiguous boundaries. The loss of spatial information exacerbates this issue, causing blurred edges and higher misclassification rates in these regions.

**Figure 6:**
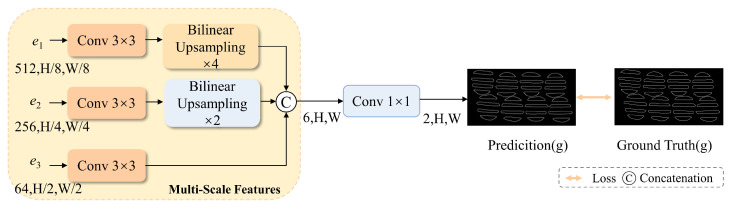
Schematic representation of the Boundary-Guided Learning Module (BLM), where the feature maps $e_{1}$, $e_{2}$, and $e_{3}$ are extracted from the backbone network ResNet50.

To address these challenges, a BLM was developed to predict boundary information. The predicted boundary maps are compared with the ground truth boundary labels to compute a boundary-specific loss. The loss ensures the model captures boundary-related features effectively through backpropagation. The structure of the BLM module is shown in Figure 6.

The BLM extracts three feature maps, $e_{1}$, $e_{2}$, and $e_{3}$, which correspond to the outputs of the first three layers of the ResNet50 backbone network. Each feature map is processed through 3 × 3 convolution operations. The first two feature maps are upsampled using linear interpolation with scaling factors of 4 and 2, respectively. This ensures consistent spatial resolution before concatenation with the third feature map. Then, a 1 × 1 convolution layer is applied to adjust the channel dimension of the feature maps at different resolutions. This operation helps unify the representation of features across different scales.

### 3.3. BatchFormerV2

To improve the model’s generalization for fiber-reinforced composites with varying fiber volume fractions, the BatchFormerV2 module [35] was introduced. Unlike conventional attention mechanisms that operate along spatial or channel dimensions, BatchFormerV2 functions along the batch dimension. It uses an attention mechanism to learn relationships between samples within a mini-batch. Specifically, for each spatial position (e.g., pixels or patches in the feature map), BatchFormerV2 treats features at the same spatial positions across all samples as a sequence. It uses Transformer-based attention to extract common features from training data with a fiber volume fraction of 60%, avoiding overfitting to individual samples while capturing greater variability in the data distribution. This enhances the model’s ability to generalize to unseen distributions, such as data with a fiber volume fraction of 54%.

The implementation of BatchFormerV2 is as follows: For each spatial position i=1,…,N, the features at the *i*-th position from all samples in the mini-batch are organized as a sequence of length *B*, where *B* is the batch size. Let $F_{i}\in R^{B\times C}$ represent the features at the *i*-th spatial position across the batch. Here, *C* is the number of channels in the feature map, and N=H×W corresponds to the total number of patches determined by the *H* and *W* of the input. BatchFormerV2 processes these patch features along the batch dimension, producing refined outputs $F_{i}^{\prime }\in R^{B\times C}$.

The mini-batch is split into two branches during training. Both branches share the same network parameters. One branch incorporates BatchFormerV2 to process the sequences ${\left\{F_{i}\right\}}_{i=1}^{N}$. The other branch bypasses BatchFormerV2 to maintain computational efficiency. BatchFormerV2 is deactivated during testing. This prevents inconsistencies from batch dependencies.

### 3.4. Loss Function

The network was trained using a custom loss function with two components: one designed to capture the overall layout of the fabric and the other to improve boundary learning. This dual-purpose approach aims to allow the model to segment fabric regions accurately and improve precision in distinguishing boundary features.

For the entire fabric image, the loss function combines the Binary Cross-Entropy (BCE) loss [36] and the Dice loss [37]. The formulation is as follows:

(2) $$L_{\mathrm{BCE}}=-\frac{1}{N}\sum_{i=1}^{N}\left(g_{i}\log\left(p_{i}\right)+\left(1-g_{i}\right)\log\left(1-p_{i}\right)\right)$$

(3) $$L_{\mathrm{Dice}}=1-\frac{2\sum_{i=1}^{N}g_{i}p_{i}+\epsilon }{\sum_{i=1}^{N}g_{i}+\sum_{i=1}^{N}p_{i}+\epsilon }$$

The parameters and variables are defined as follows: ϵ, set to $1\times 10^{-5}$, is a smoothing parameter to avoid division by zero; *g* denotes the ground truth annotation; *p* denotes the predicted annotation; and *N* is the sample count.

The BCE loss function is widely used in semantic segmentation tasks. However, the dataset in this study exhibits significant class imbalance, with foreground pixels representing fiber tows being substantially outnumbered by background pixels. This imbalance can cause the model to favor the background during training, reducing its effectiveness in segmenting fiber bundles. To address this issue, the Dice loss was incorporated alongside the BCE loss. The Dice loss enhances alignment between predicted and actual regions, especially for small foreground areas. To handle the gradient instability associated with the Dice loss, the weight for the BCE loss was set to 0.2, and the weight for the Dice loss was set to 0.8. These weights were determined through grid search to balance segmentation accuracy and optimization stability.

For effective boundary learning, it is crucial to ensure that the learned boundary features are distinct from both the background and fiber bundles. To address the severe class imbalance of boundary pixels, a weighted Binary Cross-Entropy (W-BCE) loss function was employed. The W-BCE loss is formulated as follows:

(4) $$L_{W-\mathrm{BCE}}=-\frac{1}{N}\sum_{i=1}^{N}w_{i}\left(g_{i}\log\left(p_{i}\right)+\left(1-g_{i}\right)\log\left(1-p_{i}\right)\right)$$

The parameters and variables are defined as follows: $w_{i}$ is the weight assigned to pixel *i*, which emphasizes the importance of certain pixels (e.g., boundary pixels). After conducting a grid search, the weight for boundary pixels is set to 0.8, while that for background pixels is set to 0.2. The complete formulation of this composite loss is presented as follows:

(5) $$Loss=0.2L_{\mathrm{BCE}}+0.8L_{\mathrm{Dice}}+0.005L_{W\text{-}\mathrm{BCE}}$$

### 3.5. Performance Metrics

This study employs three metrics to evaluate the performance of the model and the enhanced algorithm: Dice coefficient, Mean Intersection over Union (MIoU), and Hausdorff Distance (HD). These metrics are used consistently across all experimental analyses. The Dice coefficient and MIoU specifically measure the overlap between predicted and ground truth pixels [38]. Their mathematical definitions are as follows:

(6) $$\mathrm{Dice}=\frac{2\sum_{i=1}^{N}g_{i}p_{i}}{\sum_{i=1}^{N}g_{i}+\sum_{i=1}^{N}p_{i}}$$

(7) $$\mathrm{MIoU}=\frac{1}{N+1}\sum_{i=0}^{N}\frac{TP}{FN+FP+TP}$$

where True Positive (TP) refers to the number of pixels correctly classified as fiber bundles, True Negative (TN) denotes the number of pixels correctly classified as background, False Negative (FN) represents the number of fiber bundle pixels misclassified as background, and False Positive (FP) is the number of background pixels misclassified as fiber bundles.

The bidirectional Hausdorff Distance H(A,B) quantifies the similarity between two sets. It measures the maximum distance from a point in one set to its nearest point in the other. This effectively captures their morphological differences:

(8) H(A,B)=max(h(A,B),h(B,A))

(9) $$h\left(A,B\right)=\max_{a\in A}\{\min_{b\in B}∥a-b∥\}$$

(10) $$h\left(B,A\right)=\max_{b\in B}\{\min_{a\in A}∥b-a∥\}$$

The symbols h(A,B) and h(B,A) represent the unidirectional Hausdorff Distances from set *A* to set *B* and from set *B* to set *A*, respectively. The notation $\left|\cdot \right|$ denotes the norm used to measure the distance between the point sets *A* and *B*.

In segmentation tasks, the 95th percentile Hausdorff Distance (HD95) is commonly used instead of the maximum distance to reduce sensitivity to outliers. HD95 calculates the largest distance from a point in one set to its nearest point in the other. It excludes the most extreme 5% of distances. This metric offers a more robust evaluation of boundary differences, making it particularly suitable for segmentation tasks where noise or artifacts may introduce outliers.

### 3.6. Segmentation of Connected Yarns

Connected component analysis was performed on the predicted annotations using a four-connected region criterion. A pixel was considered connected to its neighbors if they shared one of the four cardinal directions (up, down, left, or right). This method ensured that only directly adjacent pixels were grouped into the same region. A two-pass algorithm was then employed to identify and label the connected fiber bundles.

**Figure 7:**
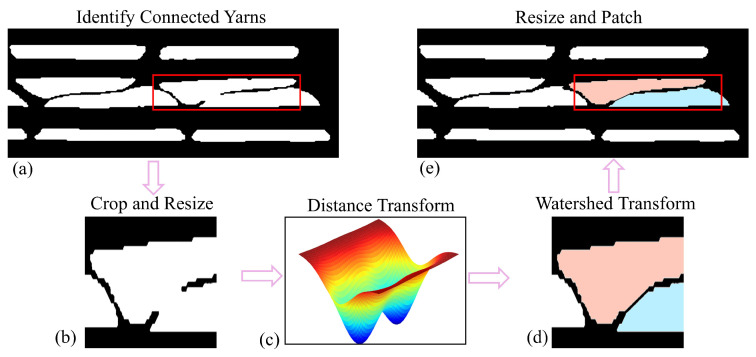
Step-by-step demonstration of the process to separate connected yarns: (**a**) detection of connected yarns, (**b**) cropping and resizing of the regions, (**c**) implementation of the Euclidean distance transform, (**d**) application of the watershed algorithm, and (**e**) resizing and restoring the original image.

In the initial pass, some connected regions might be mistakenly assigned multiple labels. To resolve these inconsistencies, a secondary scanning algorithm was applied to rescan the regions, ensuring that each region was correctly and consistently labeled. As shown in Figure 7, if the number of detected labels was fewer than the actual fiber bundles, it indicated improper separation due to inaccurate pixel grouping. Detected images with connected fiber bundles were cropped, and the cropped regions were adjusted into squares by matching their width and height, simplifying downstream processing.

To enhance feature separation, a Euclidean distance transform was applied to the resized images. This was followed by the watershed algorithm [39], which effectively isolated the connected yarns. Finally, the separated yarns were resized to their original dimensions and restored to their initial positions.

## 4. Results and Discussion

### 4.1. Fabric Structural Characteristics and Model Architecture

**Figure 8:**
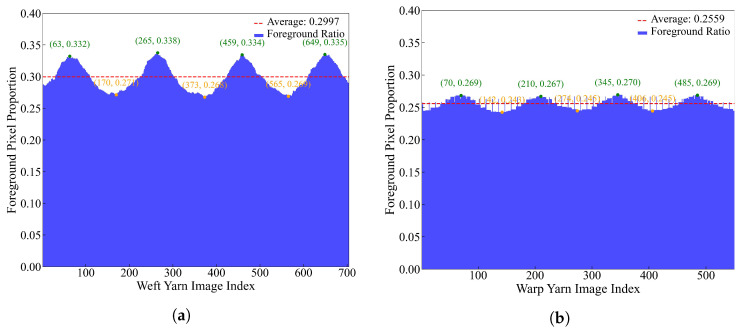
(**a**) Proportion of foreground pixels in the weft direction and (**b**) proportion of foreground pixels in the warp direction relative to the total pixels at $V_{f}=60\%$.

Figure 8 highlights a significant difference in the pixel ratios in the warp and weft directions of the fabric with $V_{f}=60\%$. It also emphasizes the distinct periodic characteristics of the fabric structure. The proportion of foreground pixels in the weft direction exhibits pronounced fluctuations, primarily due to the compaction effect of the tightly packed warp fiber tows during the interweaving process. This compression restricts the spatial distribution of the weft fibers, resulting in a reduction in foreground pixels. In contrast, the warp fibers display a more regular and stable arrangement. Their longitudinal continuity and prioritized placement during the weaving process contribute to a uniform distribution, minimizing the influence of the weft fibers on their alignment. Similar patterns were observed in the fabric with $V_{f}=54\%$. These structural characteristics, particularly the pronounced periodicity and local fluctuations, can aid in determining the size of the Representative Volume Element (RVE), which is critical for accurately capturing the fabric’s overall properties.

In this study, the weft yarns, which exhibit greater fluctuations, were selected as the primary target for segmentation. The irregular distribution of foreground pixels in the weft direction increases the complexity of boundary detection and requires the model to accurately capture intricate and ambiguous edges. Additionally, the periodic structure of the fabric demands a model capable of addressing both local details and global patterns to accurately represent fiber distribution at multiple scales. Therefore, the segmentation method was designed with three key objectives: first, improving boundary segmentation modules to enhance the model’s ability to capture complex edge features; second, strengthening generalization capabilities to ensure robust performance on unseen samples; and third, integrating multi-scale features to balance the extraction of local details with the modeling of global structures, thereby optimizing segmentation accuracy.

### 4.2. Comparative Performance Analysis of Network Architectures

To evaluate the proposed network architecture, MBL-TransUNet was compared with several well-established models. The models included FCN [14], UNet [16], UNet++ [40], and DeepLabV3+ [22]. These comparisons analyzed the segmentation performance of each model on reinforced fabric, providing a basis for further evaluation.

#### 4.2.1. Training Iterations

All models were trained using identical hyperparameter settings. During training, the learning rate (lr) was initialized at ${\mathrm{lr}}_{\mathrm{base}}=0.01$ and dynamically reduced according to the following formula:

(11) $$\mathrm{lr}={\mathrm{lr}}_{\mathrm{base}}\times {\left(1.0-\frac{{\mathrm{num}}_{\mathrm{iter}}}{{\mathrm{iterations}}_{\max}}\right)}^{0.9}$$

where ${\mathrm{num}}_{\mathrm{iter}}$ represents the current iteration number, and ${\mathrm{iterations}}_{\max}$ indicates the total number of iterations for training.

A batch size of 4 was used. The Stochastic Gradient Descent (SGD) optimizer [41], configured with a momentum of 0.9 and a weight decay of 0.0001, was employed to update the model weights. The lr was dynamically adjusted by automatically updating the lr in the optimizer’s parameter groups. This ensured precise control over the learning process.

**Figure 9:**
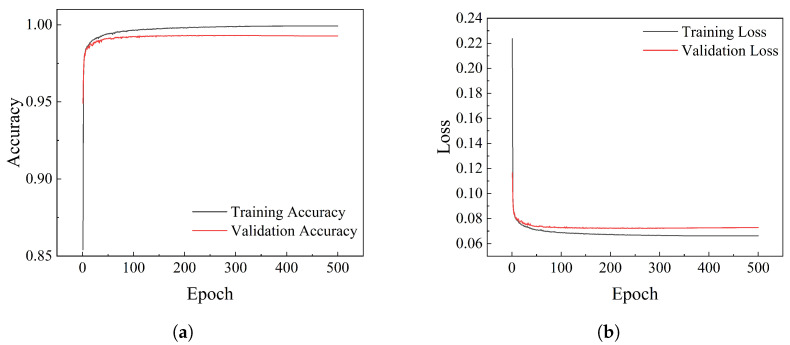
(**a**) Depiction of the loss trajectory throughout the training and validation phases. (**b**) Representation of the accuracy trajectory observed during the training and validation phases.

To track loss and accuracy metrics across epochs, an early stopping strategy was avoided. This decision prevented premature termination of the training process. After 500 epochs, the loss and accuracy curves, shown in Figure 9, indicate gradual convergence of the training process over time. Only the training loss and accuracy curves for the MBL-TransUNet model are displayed here, while the corresponding curves for other models are excluded.

#### 4.2.2. Training Dataset Results Analysis

**Figure 10:**
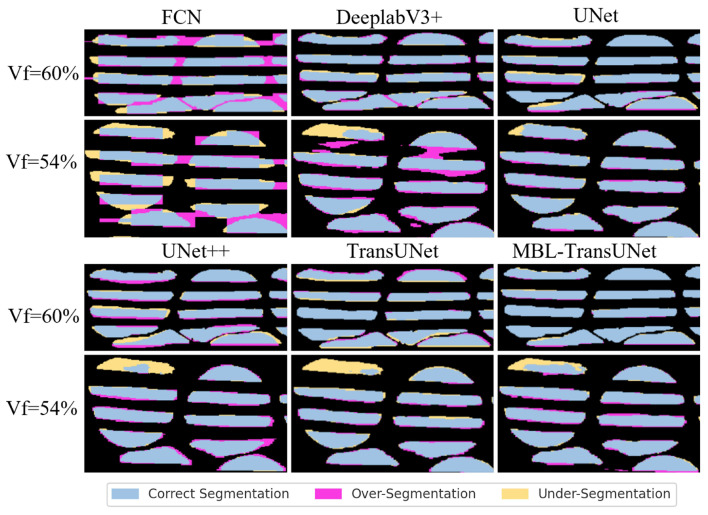
The comparison of segmentation performance between the MBL-TransUNet model and other classical models is demonstrated.

The dataset with $V_{f}=60\%$ was used for both training and testing the model. Qualitative analysis was conducted first on this dataset to evaluate the performance of the proposed method. The results from each network were visualized to highlight the differences. As shown in Figure 10, FCN and DeepLabV3+ exhibit segmentation inaccuracies. UNet, UNet++, and TransUNet demonstrate under-segmentation in critical areas. In contrast, the proposed MBL-TransUNet model effectively captures intricate details. It maintains precision in complex regions, demonstrating superior stability and segmentation accuracy.

**Figure 11:**
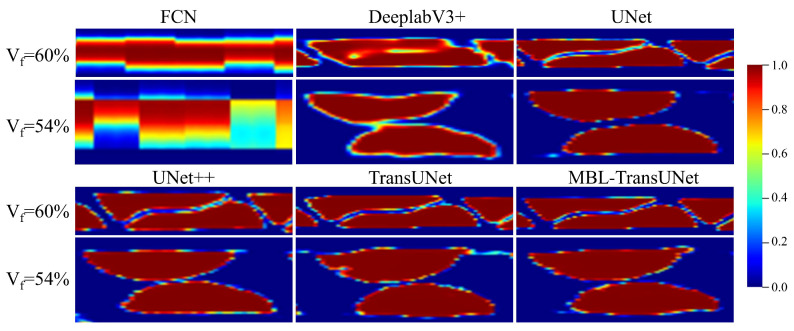
The heatmaps generated by the MBL-TransUNet model and those produced by alternative models.

The heatmaps in Figure 11 vividly illustrate the activation levels predicted by different models. The color scale represents the confidence in pixel classification. Red indicates high activation values, showing strong confidence that the pixel belongs to the foreground category. Blue represents low activation values and shows high confidence that the pixel belongs to the background. Yellow or orange denotes moderate activation, reflecting uncertainty in distinguishing between foreground and background. Green or light blue typically appears in boundary regions, highlighting areas of ambiguity between the two classes.

The heatmaps reveal notable differences in activation patterns across models. A comparison highlights these differences in detail. The FCN model produces sparse and irregular activations, with large yellow and orange areas near boundaries. These indicate significant uncertainty and poor boundary detection. DeepLabV3+ demonstrates slightly more consistent activations. However, it continues to struggle with boundary precision, as seen in fluctuating activations and residual uncertainty. UNet and UNet++ exhibit greater stability in their activation patterns, with fewer uncertain areas. Medium activation values remain near edges, limiting their ability to capture intricate boundary details. TransUNet improves boundary delineation and overall activations, though some uncertainty persists in complex regions. In contrast, the MBL-TransUNet model significantly outperforms all other networks. Its activation values (red areas) closely align with ground truth boundaries. The boundary regions are sharply defined with minimal green or light blue areas. This exceptional capability highlights MBL-TransUNet’s effectiveness in addressing complex boundary issues and achieving precise segmentation.

**Figure 12:**
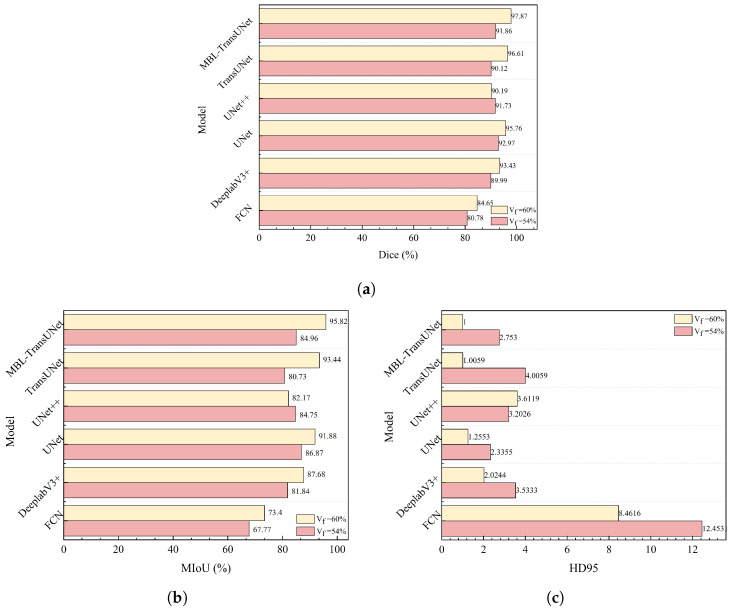
A comparative analysis of segmentation performance on composite fabric images using different network architectures. (**a**) The Dice of segmentations from different networks. (**b**) The MIoU of segmentations from different networks. (**c**) The HD95 of segmentations from different networks.

The quantitative analysis results are shown in Figure 12. On the dataset with $V_{f}=60\%$, the proposed MBL-TransUNet model demonstrated superior performance across all evaluated metrics. Specifically, it outperformed UNet by 2.11% in the Dice coefficient and 3.94% in the MIoU, while reducing the HD95 by 0.26%. The proposed method also showed improvements over TransUNet in all metrics. It achieved gains of 1.26% in the Dice coefficient and 2.38% in the MIoU.

#### 4.2.3. Zero-Shot Results Analysis

The dataset with $V_{f}=60\%$ was used to train the model, while the $V_{f}=54\%$ dataset was employed for testing. A zero-shot analysis evaluated the model’s generalization ability on unseen data. It provided insights into the model’s performance with previously unencountered data.

Qualitative analysis of $V_{f}=54\%$ samples showed trends consistent with those from $V_{f}=60\%$. Figure 10 and Figure 11 illustrate these observations across most models. However, for UNet and MBL-TransUNet, the differences were less visually apparent.

Quantitative analysis, illustrated in Figure 12, revealed that MBL-TransUNet was slightly less robust than UNet. Specifically, UNet outperformed MBL-TransUNet by 1.11% in the Dice coefficient and 1.91% in the MIoU. However, MBL-TransUNet achieved a lower HD95 by 0.42, indicating better boundary detection.

When compared to TransUNet, the proposed method demonstrated superior performance across all metrics, further highlighting its robustness and generalization. UNet achieved the best results at $V_{f}=54\%$ but performed poorly at $V_{f}=60\%$. This highlights the challenge of balancing robustness and performance, which remains a focus for future research.

In this experiment, the model exhibited improved generalization when trained on images with $V_{f}=60\%$ and evaluated on zero-shot images with $V_{f}=54\%$. Conversely, when the training utilized $V_{f}=54\%$ images and testing was conducted on $V_{f}=60\%$ images, the model’s generalization capabilities diminished. This variation can be attributed to the role of volume fraction on fiber structure as well as the model’s learning dynamics. In $V_{f}=60\%$ samples, the fibers’ relative positions and interlacing patterns exhibit greater stability, enabling the model to learn the interlacing patterns of fiber bundles at both local and global scales, thus achieving a broader structural representation. While these structural characteristics apply to $V_{f}=60\%$ samples, they can also accommodate the structural variations found in lower volume fraction ($V_{f}=54\%$) samples. A higher volume fraction signifies an increased fiber density within the samples, which allows the model to capture a wider array of feature patterns and global structural insights. Although these patterns may differ in $V_{f}=54\%$ samples, the overarching features and intricacies acquired from high volume fraction training continue to aid in processing samples with lower volume fraction. Conversely, lower volume fraction samples display a more dispersed fiber arrangement. When the model is trained on $V_{f}=54\%$ samples, it concentrates mainly on local structural features, thereby lacking the global adaptability necessary for comprehending high-density fiber configurations. Consequently, training on $V_{f}=54\%$ hinders the model’s ability to generalize to $V_{f}=60\%$.

Moreover, it is crucial to acknowledge that the present model is inadequate for segmenting distinct types of fabric. The variations in weaving patterns and fiber arrangements necessitate an enhancement in the model’s capacity for generalization across a variety of fabrics. Consequently, for the precise segmentation of various fabric types, the model necessitates further optimization and must be specifically trained for each individual fabric category.

### 4.3. Module Ablation Study on Segmentation Performance

The proposed MBL-TransUNet model incorporates multiple modules, including BatchFormerV2, BLM, and MFBEM, to achieve higher segmentation accuracy, improved boundary detection, and enhanced generalization. However, the exclusion or mismatch of certain modules can significantly degrade performance. To evaluate the contributions of each module and validate the effectiveness of the modular design, ablation experiments were conducted to analyze their individual and combined impacts on segmentation performance.

#### 4.3.1. Ablation Analysis of Different Modules

Table 2 summarizes the model variant components and their corresponding segmentation performance metrics.

BatchFormerV2 enhances the model’s generalization capabilities, enabling superior performance when processing materials with varying fiber volume fractions. For samples with $V_{f}=54\%$, BatchFormerV2 increased the Dice coefficient from 90.12% to 91.27%, improving it by 1.15%. MIoU rose from 80.73% to 83.96%, an increase of 3.23%. HD95 decreased significantly from 4.0059 to 3.0882, showing its effectiveness in adapting to variations in fiber volume fractions.

The BLM module improves boundary segmentation accuracy by training on boundary-specific features in fiber-reinforced materials. It reduced the HD95 value significantly, from 4.0059 to 3.6144 at $V_{f}=54\%$ and to 1.0000 at $V_{f}=60\%$. These results demonstrate its ability to capture complex boundary details and significantly improve segmentation precision.

Integrating the MFBEM module significantly improves the model’s performance. At $V_{f}=54\%$, MIoU rose from 80.73% to 80.91%. HD95 decreased by 0.3% compared to TransUNet. At $V_{f}=60\%$, the Dice coefficient improved from 96.61% to 97.13%. MIoU increased from 93.44% to 94.43%. These findings highlight that the MFBEM module markedly enhances the model’s segmentation capabilities across varying fiber volume fractions. Its ability to incorporate multi-scale feature representations facilitates the transfer of feature information across levels, improving the model’s ability to capture both local details and global structures. This comprehensive integration significantly refines the model’s understanding of the fabric structure, resulting in optimally segmented outcomes.

However, the combined inclusion of the BLM and MFBEM modules alone led to a substantial decline in performance. At $V_{f}=54\%$ test set, the Dice coefficient, MIoU, and HD95 dropped to 80.53%, 67.41%, and 7.4729, respectively. Similarly, at $V_{f}=60\%$ test set, these metrics decreased to 82.47%, 70.19%, and 6.1323, respectively. This performance degradation can be attributed to several factors. First, inconsistencies in optimization trajectories and conflicting feature representations between the modules hindered performance. While the BLM module focuses on learning boundary features, the MFBEM module emphasizes multi-scale feature fusion, leading to potential redundancy or conflicts in feature expression. Second, the absence of BatchFormerV2 reduced the model’s ability to perform global feature modeling, making it difficult to reconcile feature variations across training samples. This limitation predisposed the model to local optimization. Finally, the increased network complexity from the additional modules destabilized the optimization process during training. These factors collectively constrained the model’s segmentation performance, underscoring the critical role of BatchFormerV2 in improving global feature modeling, facilitating module synergy, and enhancing generalization.

The experimental results, with the best outcomes highlighted in bold, demonstrate the critical role of integrating BatchFormerV2, BLM, and MFBEM in achieving optimal segmentation performance. By effectively combining global and local features, the model excels at capturing intricate boundaries and delivers superior results across all evaluation metrics. These findings validate the effectiveness and robustness of the proposed architecture.

In summary, BatchFormerV2, BLM, and MFBEM each contribute to improving segmentation performance, while also exhibiting certain limitations. BatchFormerV2 significantly enhances the model’s generalization ability and global feature modeling but increases computational complexity and training costs. BLM optimizes boundary feature learning, improving boundary segmentation accuracy and reducing HD95. However, its strong focus on boundary regions may weaken overall segmentation performance and make it more sensitive to annotation quality. MFBEM enhances segmentation accuracy through multi-scale feature fusion but may introduce feature redundancy, increase model complexity, and reduce optimization stability in the absence of global constraints. When BLM and MFBEM were integrated without BatchFormerV2, performance degradation occurred due to feature redundancy, lack of global feature modeling, and increased optimization instability. This highlights the critical role of BatchFormerV2 in maintaining feature consistency, facilitating module synergy, and improving generalization. The experimental results (highlighted in bold) further confirm that the integration of BatchFormerV2, BLM, and MFBEM effectively balances global and local features, achieving superior segmentation performance.

#### 4.3.2. Ablation Analysis of MF and BE Modules in MFBEM

The ablation results in Table 3 highlight the contributions of the MF and BE modules to the model’s segmentation performance, detailing the individual and combined effects of these components.

At $V_{f}=60\%$, the MF module significantly improves performance, increasing the Dice coefficient from 96.61% to 97.10% and the MIoU from 93.44% to 94.38%. This demonstrates the effectiveness of multi-scale feature aggregation in enriching semantic representation and enhancing the capture of small target boundaries in fiber segmentation. However, at $V_{f}=54\%$, the robustness of the MF module declines sharply. The Dice coefficient drops from 90.12% to 74.01%, and the HD95 increases to 15.7287. This substantial decline reveals the limitations of the multi-scale fusion approach, suggesting a trade-off between capturing intricate details and maintaining robustness. The pronounced feature differences between the two datasets indicate that the fusion patterns effective for $V_{f}=60\%$ cannot be directly applied to $V_{f}=54\%$, despite the consistency in the weaving types of the fibers.

The BE module significantly improves boundary accuracy in both $V_{f}=60\%$ and $V_{f}=54\%$ scenarios, though the gains are slightly smaller for $V_{f}=54\%$. At $V_{f}=54\%$, the Dice coefficient increases from 90.12% to 90.71%, with HD95 reduced to 3.4241. At $V_{f}=60\%$, BE enhances the Dice coefficient and MIoU to 97.23% and 94.62%, respectively.

Combining the MF and BE modules is a logical step to maximize performance. Both modules contribute positively to segmentation accuracy, with their combined configuration (TransUNet + MF + BE) delivering particularly strong results. Specifically, this configuration achieves Dice and MIoU values of 91.86% and 84.96% for $V_{f}=54\%$, with HD95 reduced to 2.7530. For $V_{f}=60\%$, Dice and MIoU reach 97.87% and 95.82%, with HD95 reduced to 1.0000. These findings indicate that applying multi-scale feature fusion before boundary refinement enables the BE module to capture precise boundary details more effectively, whereas reversing the order struggles to mitigate noise introduced by multi-scale processing. The integration of multi-scale feature fusion from the MF module with the boundary refinement capabilities of the BE module results in optimal segmentation accuracy. This combination enhances robustness and ensures precise boundary alignment, addressing the challenges of intricate fiber segmentation.

## 5. Conclusions

Fiber-reinforced composite materials possess complex multi-scale structures. Micro-CT technology, renowned for its high-resolution three-dimensional imaging capabilities, effectively captures the internal configurations of composite materials, providing crucial data for digital twin modeling. In this study, a preform consisting of eight plies of glass non-crimp fabric was scanned using Micro-CT. Due to the intricate weaving structure of the fiber fabric and indistinct interfaces, conventional image processing methods encounter difficulties in accurately segmenting the fiber bundle interfaces. While traditional image processing software performs excellently in segmenting clear boundaries, its accuracy decreases when confronted with complex woven regions. To address this issue, this study proposes the MBL-TransUNet model, which leverages deep learning techniques to enhance segmentation precision in woven fiber areas, providing a more accurate solution for material property prediction and digital twin modeling of composite materials. The model integrates three key modules—BatchFormerV2, BLM, and MFBEM—and outperforms traditional network architectures, such as FCN, UNet, UNet++, and DeepLabV3+, demonstrating significant advantages in segmentation accuracy for this task.

The ablation experiments emphasize the contribution of each individual module. BatchFormerV2 enhances synergy among modules and improves generalization capabilities. At $V_{f}=54\%$, it increases the Dice coefficient from 90.12% to 91.27%, MIoU from 80.73% to 83.96%, and reduces HD95 from 4.0059 to 3.0882. The BLM module significantly improves boundary segmentation accuracy, decreasing HD95 from 4.0059 to 2.7530 at $V_{f}=54\%$, and further reducing it to 1.0000 at $V_{f}=60\%$. The MFBEM module enhances multi-scale feature fusion, increasing MIoU from 80.73% to 80.91% at $V_{f}=54\%$, and from 93.44% to 94.43% at $V_{f}=60\%$.

The combined effect of all three modules delivers the best segmentation performance. At $V_{f}=54\%$, the Dice coefficient, MIoU, and HD95 achieved 91.86%, 84.96%, and 2.7530, respectively, reflecting improvements of 1.74% in Dice, 4.23% in MIoU, and a reduction of 1.25 in HD95 compared to the baseline model. At $V_{f}=60\%$, the three metrics reached 97.87%, 95.82%, and 1.0000, with Dice and MIoU improving by 1.26% and 2.38%, and HD95 reducing to 1.0000.

It is noteworthy that the performance significantly degraded when only BLM and MFBEM were integrated without BatchFormerV2. At $V_{f}=54\%$, Dice and MIoU decreased to 80.53% and 67.41%, respectively, while HD95 increased to 7.4729. Similar drops were observed at $V_{f}=60\%$. BLM focuses on boundary features, while MFBEM emphasizes multi-scale feature fusion. The integration of these modules led to redundancy and inconsistent feature representations. BatchFormerV2 addressed these issues by enabling cross-batch learning, which enhanced global feature modeling and ensured better synergy between the modules.

The proposed model has demonstrated exceptional performance in segmenting eight plies of glass non-crimp fabric. Future research could further explore the model’s generalization capabilities, particularly its segmentation performance in zero-shot semantic segmentation tasks. Additionally, optimizing the network to effectively handle more complex fabric structures, such as varying weave patterns and fiber types, presents another promising direction for future research. 

## Figures and Tables

**Table 1:** Comparison of Advantages and Disadvantages of Composite Fabric Image Segmentation Algorithms.

Algorithm	Advantages	Disadvantages
Traditional	Requires less training data, computationally efficient.	Poor for complex segmentation tasks, sensitive to noise and complex backgrounds.
FCN	Fast computation, simple model structure.	Loses detail information, sensitive to noise, and lower resolution.
U-Net	Captures global and local information, retains image details well.	Less effective for targets with significant size variations.
DeepLab V3+	Handles large images with high segmentation accuracy.	Handles blurred edges poorly, complex model leads to longer training.
TransUNet	Combines Transformer’s global context modeling with U-Net’s low-level feature extraction.	High complexity results in slower training and inference speeds.

**Table 2:** Ablation experiment: the impact of each module on the model segmentation performance.

Model	Components				$V_{f}=54\%$			$V_{f}=60\%$		
	Trans-UNet	BatchformerV2	BLM	MFBEM	Dice (%)	MIoU (%)	HD95	Dice (%)	MIoU (%)	HD95
0	✓				90.12	80.73	4.0059	96.61	93.44	1.0059
1	✓	✓			91.27	83.96	3.0882	97.11	94.38	1.0143
2	✓		✓		90.40	82.50	3.6144	97.12	94.41	**1.0000**
3	✓			✓	89.43	80.91	4.0019	97.13	94.43	1.2351
4	✓	✓	✓		90.14	82.06	3.4561	96.99	94.17	1.0059
5	✓	✓		✓	91.10	83.66	3.2192	97.12	94.41	1.1231
6	✓		✓	✓	80.53	67.41	7.4729	82.47	70.19	6.1323
7	✓	✓	✓	✓	**91.86**	**84.96**	**2.7530**	**97.87**	**95.82**	**1.0000**

**Table 3:** Ablation experiment: the impact of the MFBEM on the model segmentation performance.

Model	$V_{f}=54\%$			$V_{f}=60\%$		
	Dice (%)	MIoU (%)	HD95	Dice (%)	MIoU (%)	HD95
TransUNet	90.12	80.73	4.0059	96.61	93.44	1.0059
TransUNet + MF	74.01	59.11	15.7287	97.10	94.38	1.0143
TransUNet + BE	90.71	83.00	3.4241	97.23	94.62	1.0059
TransUNet + BE + MF	91.60	84.51	2.9130	97.14	94.44	**1.0000**
TransUNet + MF + BE	**91.86**	**84.96**	**2.7530**	**97.87**	**95.82**	**1.0000**

## Data Availability

The data are contained within the article.
